# Understanding Allogrooming Through a Dynamic Social Network Approach: An Example in a Group of Dairy Cows

**DOI:** 10.3389/fvets.2020.00535

**Published:** 2020-08-04

**Authors:** Inés de Freslon, J. M. Peralta, Ana C. Strappini, Gustavo Monti

**Affiliations:** ^1^Faculty of Veterinary Sciences, Preventive Veterinary Medicine Institute, Universidad Austral de Chile, Valdivia, Chile; ^2^College of Veterinary Medicine, Western University of Health Sciences, Pomona, CA, United States; ^3^Faculty of Veterinary Sciences, Animal Science Institute, Universidad Austral de Chile, Valdivia, Chile

**Keywords:** dairy cows, social grooming, stochastic actor-oriented models, dynamic network analysis, social behavior

## Abstract

For gregarious species such as domestic cattle, the social environment is a very important determinant of their welfare and fitness. Understanding the complexity of cows' relationships can assist the development of management practices that are more integrated with the cows' social behavioral processes. The two aims of this study were: (1) to determine the dynamics of affiliative relationships, as indicated by allogrooming, by means of stochastic actor-oriented modeling, in dairy cows during early lactation; (2) to explore the underlying processes and the individual attributes, such as age, social rank and reproductive state, that could shape network pattern changes in grooming contacts between individual. We observed the allogrooming behavior of a dynamic group of 38 dairy cows for 4 h per day for 30 days. Using stochastic actor-oriented models, we modeled the dynamics of weekly contacts and studied how structural processes (e.g., reciprocity, transitivity, or popularity) and individual attributes (i.e., age, social rank, and reproductive state) influence network changes. We found that cows tended to groom individuals that had previously groomed them, implying a possible cooperation. Cows that groomed more actively did not appear to have a preference for specific individuals in the herd, and in return, tended to be groomed by fewer cows over time. Older individuals groomed more cows than younger ones, indicating that allogrooming could be related to seniority. Cows groomed mainly individuals of similar age, suggesting that familiarity and growing up together enhanced social grooming. Over time, cows with higher social rank were groomed by fewer cows and individuals recently reintroduced to the group groomed more herdmates. The study of social network dynamics can be used to better understand the complexity and non-linearity of cow relationships. Our findings, along with further research, can complement and strengthen the design of improved management practices that are more in line with the natural social behavior of cows.

## Introduction

Dairy cows are herding animals that thrive in socially stable groups. Living in a herd serves as protection against predators (1) but also entails competition for resources and the need for a social organization and group stability. Cattle by nature develop matrilineal social systems through affiliative, cooperative, and agonistic behaviors (2, 3) and also form long-lasting preferential relationships (4, 5). In modern dairy production systems, cow herds are usually divided into multiple groups to facilitate management and they are commonly arranged according to the stage of lactation and nutritional requirements. Cows are moved to different pens or paddocks for breeding, treatment, and other management practices. Essentially, depending on their physiological and production state, each cow is relocated to a new area where she is housed with different herd mates and subject to the appropriate management routines (6). One of the most critical management practice in terms of cow welfare is the transition period, which is generally 3 weeks pre- until 3 weeks post-calving. During this relatively short period, cows are frequently regrouped, and exposed to several stressors like calving, separation from the calf, diet changes and the onset of lactation. During regrouping, cows must re-establish their social structure and incoming individuals must also recover from the breakup of their former social bonds and adapt to a new social environment. The negative impact of regrouping on behavior, animal welfare, and productivity has been well-documented. Several studies have demonstrated an increase in agonistic behaviors (7), as well as decreases in affiliative interactions and resting periods, feeding and rumination times, and milk production (8–11). A better understanding of the ability of dairy cows to adapt to a changing social environment is needed (5, 12).

Allogrooming behavior, also called social grooming, serves a variety of functions in cattle. Besides its hygienic utility (13) and the provision of pleasure (14, 15), it is also known to serve several social purposes. Inter-individual bonds and preferential relationships are mainly established, maintained, and reinforced through allogrooming (13, 14, 16, 17). This behavior also enhances group cohesion and maintains social stability, reduces social tension and has calming effects (13, 14, 16, 18). Given the important functions of social grooming, this behavior could be used to evaluate social stability and maintenance of social bonds in cows.

Social network analysis (SNA) has emerged as a powerful tool to study animal interactions and relations (19). This method goes beyond the study of dyadic interactions, and includes the influence of third parties, allowing for the analysis of interactions at an individual, dyadic, and group level (20). Thus, SNA facilitates the understanding of complex social patterns, analyzing not only how individual behavior contributes to the general structure of the group, but also how group structure influences dyadic interactions.

There is a current need to take into account temporal aspects of contact structure in animal social network analysis (21, 22). Direct and indirect interactions between group members are dynamic. These dynamic interactions are shaped not only by individual characteristics and environmental factors but also by individuals joining or leaving the group (23, 24). Analyzing social data through dynamic SNA provide information on how the network evolves over time and how resilient it is to disturbances that can affect the group (25, 26). A few statistical modeling methods for dynamic networks have been developed, but their application in animal studies is still very limited [for reviews see (22, 27)]. One of these methods, stochastic actor-oriented modeling (SAOM), have been signaled as a promising tool for analyzing social network dynamics. This analytical instrument studies how individual attributes and network processes determine the probability of individuals to interact or associate with each other over time (22). The model can include multiple covariates at individual and/or dyadic level, that can be fixed or, if they change over time, dynamic. SAOM can take into account changes in the composition of the group, if some individuals join or leave the group during the process (28). Overall, the SAOM framework enables to study how individuals change their relationships in response to the present network structure, i.e., group behavior (29). Despite its strong potential, SAOMs have been less explored in animal studies compared to social sciences studies [barbary macaques: (26); black-capped chickadees: (30); bottlenose dolphins: (31); crickets: (27, 32); farmed salmons: (33); fruit flies: (34); rooks: (35); spiders: (36); spotted hyenas: (24); vervet monkeys: (37)].

To date, several studies have characterized social networks in cattle, using association networks based on spatial proximity data [(12, 38–46)]. Although very valuable, association networks only provide information based on potential contacts or relations. Contact networks, based on direct or indirect observations, can provide more detailed information as for example the type of interaction and the directionality (who starts the interaction and who receives it). Despite the major potential for social grooming networks to provide information on the dynamic of social behavior, those specific networks have been rarely explored in cattle (47, 48).

The main two aims of this study were: (1) to investigate network dynamics of affiliative relationships using allogrooming as an indicator in dairy cows during early lactation; (2) to explore the underlying processes and the individual attributes (such as age, social rank, and reproductive state) that could shape network pattern in grooming contacts between individuals.

## Materials and Methods

### Study Design

**Figure 1:**
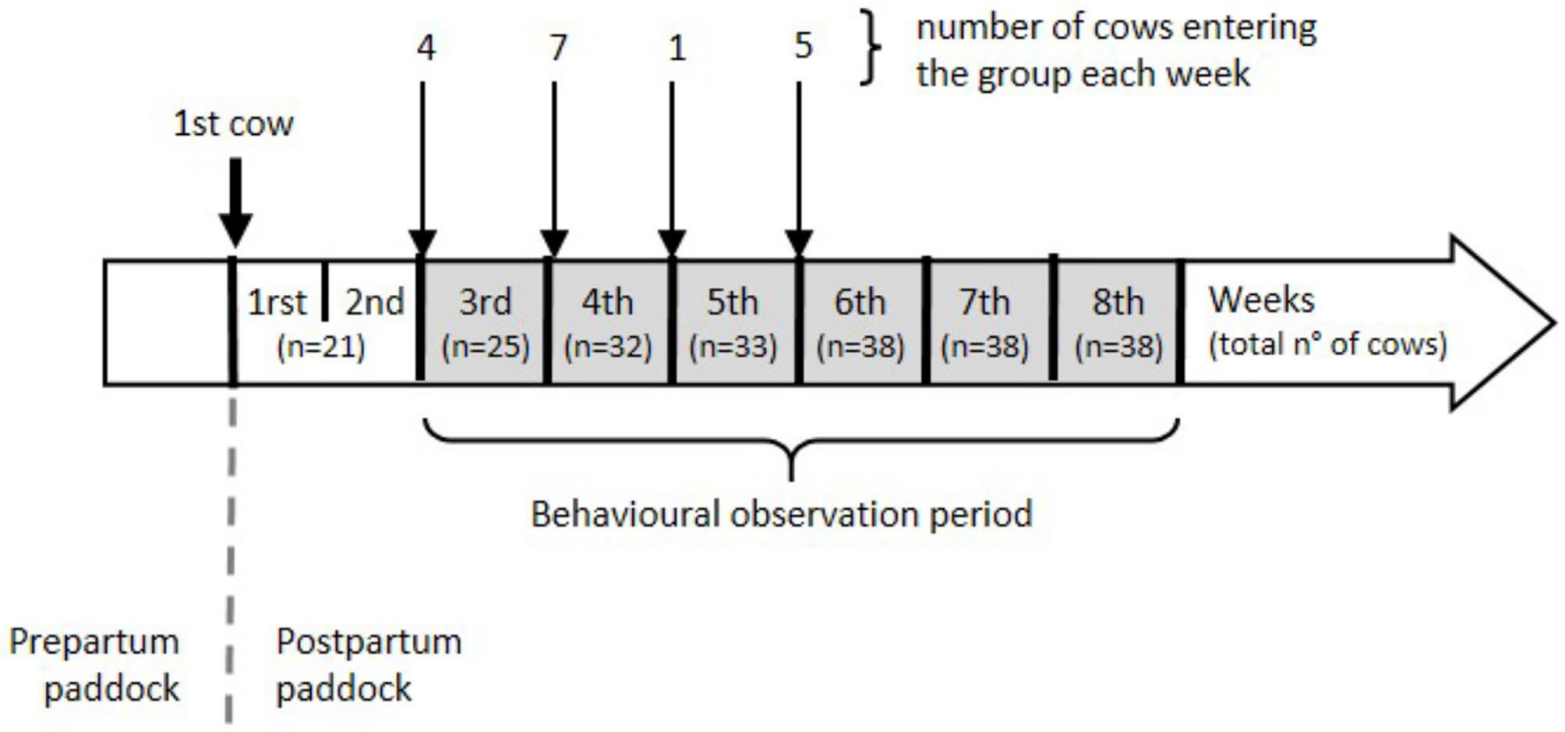
Study timeline.

We conducted the study during the months of February and March at a pasture-based dairy farm of the Agricultural Research Station of Universidad Austral de Chile, located in Valdivia (39°47'S, 73°13'W). We selected from the same herd a total of 40 Holstein dairy cows with similar expected parturition date. Those individuals were between 2.5 and 10.2 (mean = 4.5, S.D. = 1.9) years of age and were all born in the farm and grown as replacement heifers on site. We took care not to include mothers and daughters in the same experimental group to avoid potential biases due to other type of bonds. Cows were enrolled in the study 2 months before starting the experimental procedures and accommodated in a 1-acre prepartum paddock. Calving took place in the same paddock, and each calf was separated from the dam less than 24 h postpartum, which is a standard practice in dairy farms. After separation, each cow was moved to the 1.4-acre study paddock. Data collection lasted 6 weeks and started 2 weeks after the first cow entered the study paddock to allow for a sufficient number of cows to be present at the beginning of the observation period. Cows were introduced to the study group once a week, and the number of introductions depended on how many cows calved (Figure 1). No new animals were included in weeks 5 and 6. In addition to grazing, cows received silage supplementation twice a day, had *ad libitum* access to water, and concentrate feed was provided during milking. Cows were milked twice daily, in the morning and afternoon.

### Behavioral Measures

Before the beginning of the behavioral observations, we identified each cow with a large unique numeric symbol painted with non-toxic marker on each flank (Blondor Wella®, Kronberg, Germany). In a preliminary study, we observed the group at different periods in the morning, afternoon, evening, and night. This enabled us to select the most appropriate observation schedule, based on the period the cows were more active, daylight visibility and in between regular milking times to prevent this management routine from interfering with the experimental observations. We then defined two daily observation blocks: mornings from 0900 h to 1200 h and afternoons from 1800 h to 2100 h. Each block consisted of four 30-min continuous observation periods with 20-min intervals, which resulted in a total daily observation time of 4 h. We observed the study group during 5 consecutive days per week for 6 weeks (30 days).

We continuously recorded the occurrence of allogrooming contacts, defined as a cow repeatedly using her tongue to lick any part of the body of another cow, except the ano-genital area. If a grooming bout stopped for more than 20 s or was interrupted by another behavior and then resumed, we recorded it as two grooming events. We also registered the identities of the animal that performed the behavior and the recipient. During the same observation period, we recorded episodes of headbutting behavior to calculate the social rank of each cow. We defined headbutting as a cow hitting another cow with its forehead, followed by a withdrawal of the targeted cow. Cows were familiar with human presence, which enabled the observers to watch them from a distance of at least 3 m without disturbing them as well as from a platform (3 m height).

In each session, observations were performed simultaneously by two observers, out of a group of 5 trained observers, from a platform at 3 m height. To ensure an adequate agreement among the observers, we conducted an inter-observer reliability test before starting the experiment using the same cows' group. The five observers simultaneously conducted four continuous observation periods of 30 min, in which they registered every allogrooming and headbutting events observed. We analyzed the inter-observer reliability for each behavior using the intraclass correlation coefficient. We calculated its estimates and their 95% confident intervals were based on a mean-rating (k = 5), absolute-agreement, 2-way mixed-effects model, using statistical package SPSS Statistics for Windows, version 25 (IBM Corp., Armonk, N.Y., USA). The study started once the observers obtained an ICC significant and superior to 0.8 (Allogrooming: ICC = 0.87, *p* < 0.001; Headbutting: ICC = 0.81, *p* < 0.001), indicating a good reliability (49).

#### Estimation of Social Rank

We obtained the social rank (SR) based on headbutting records, using the dominance index (DI) of Lamprecht (50). In brief, we created matrices based on weekly dyadic agonistic interactions. For each dyad, we determined the dominant cow as the one with the highest number of headbutts toward the other, which we identified as the subordinate animal. We obtained the DI of each cow by dividing the number of its subordinates by the number of animals with which she interacted. We classified cows in three groups (17): low-ranked (DI < 0.40), medium-ranked (0.40 ≤ DI < 0.60), and high-ranked (DI ≥ 0.60).

### Predictor Variables

As predictors of allogrooming, we included age, social rank, reproductive state, and date of entry. We assigned the age based on the farm records and used it as a continuous variable. We tested for the consistency of the DI across weeks using the intraclass correlation coefficient. We defined Entry as the week a cow entered the postpartum group. We coded this variable with two categories: “already present,” when the animal was present in the group at least a week before, or “newly entered,” when the animal entered the group the present week. We determined weekly reproductive state (Rstate) based on ultrasonography. When a cow displayed oestrus behavior, we separated her after milking time and scanned the ovarian structures to assess the size of the pre-ovulatory follicles. We then performed a second ultrasonography 24 h later to detect ovulation. Finally, we carried out a new ultrasound 7 days later to determine the formation of a new corpus luteum and confirm ovulation. This variable was coded with two categories: “in oestrus,” when the animal was in oestrus during the week, or “open,” when oestrus wasn't detected during that week.

### Network Construction and Characterization

We constructed allogrooming networks based on separate weekly observations. Networks were formed by nodes, i.e., cows, and the allogrooming events were the connectors or ties to one another, with the number of grooming events between two animals representing the weight of the ties. We also took into consideration the direction, to and from initiator to recipient. The main network descriptive metrics are detailed in Table 1. We also calculated for each individual the weighted outdegree/indegree, defined as the number of contacts in which the animal was the initiator/receptor, respectively. This measure was normalized based on the maximum possible degree in each network, to allow for comparison between networks of different sizes. We constructed the networks with the Igraph package for R [(51); version 3.4.3, (52)].

**Table 1:** Main network descriptive metrics (19).

**Parameter**	**Description**
Density	Ratio of the number of dyads (i.e., link between two nodes) to the number of possible dyads in the network. Its values ranged from zero to one, with one indicating a dense and fully connected network, i.e., all nodes were directly interconnected.
Average path length	Average of all the shortest paths of the network. A path is the number of steps (connectors) between two nodes. A high average path length indicates that individuals tend to connect to others very indirectly.
Diameter	Longest of all the shortest paths indicate the cohesiveness of the group. A shorter diameter usually represents a denser network.
Reciprocity	Proportion of reciprocated ties related to the total number of ties.

### Model Specification and Estimation

We modeled the allogrooming dynamic networks using stochastic actor-oriented models (SAOM) with the RSiena package [version 1.2-3, (28)]. SAOM characterizes network dynamics being driven by different predictor variables, called effects, that operate simultaneously. This model assumes that changes in the network are based on each cow's decision to groom another cow. Expressly, individuals control their own interactions and modify them to optimize their position in the network in regard to whom they interact with and when. Another assumption of the model is that changes occur on a continuous time basis between discrete time points, and the probability of change in the network's ties is the consequence of a Markov process, as the current state of the network determines probabilistically its future states (28). Between observations, each individual in a series of mini-steps can decide to form a new tie, maintain an existing tie or to dissolve it. This decision is based on their and other's attributes, their network status and their evaluation on the current network. Even though the time parameter is continuous, RSiena model needs a series of consecutive observations of the network (i.e., matrices or waves) at discrete time points but assumes that changes between those waves occur on a linear time basis. Therefore, six consecutive matrices that represent six observation time points were constructed, one for each of the 6 weeks of observation. RSiena also requires matrices to be binary as the analysis for weighted networks is not yet implemented. In order to turn our weighted networks into binary matrices, we transformed ties weight values greater than one into ones and left the rest as zeros. Thus, cows were connected in a given week if one cow groomed the other cow at any point in the week (value 1), and not connected if they did not groom during that specific week (value 0). Matrices were asymmetric as we considered the direction of the ties. We assessed the amount of change between two consecutive waves with the Jaccard index. It is a measure of stability that reflects the proportion of stable ties persisting from one wave to another compared to the total number of ties (including stable, created, and dissolved ties) (53). The value ranges from 0 (null degree of similarity between waves) to 1 (waves are exactly the same). We used the method of joiners and leavers to account for compositional changes, reflecting the number of cows entering and leaving the group at different time points, and for the proportion of time each cow was present every week (28).

SAOM effects can be structural or covariate-related [for a detailed description of all the available effects see (28)]. Covariate effects are based on predictor variables and can be constant or changing (time-varying). Since SR was stable over the weeks (ICC = 0.79, *p* < 0.001), we added it as constant actor covariate, together with Age. We incorporated Entry and Rstate as changing covariates with a different category on the week an animal joined the group or ovulated, respectively.

We first added basic structural effects, namely outdegree, reciprocity, and transitive triplets (detailed in Table 2). Afterwards we added the remaining structural effects of interest followed by actor covariates. For each actor covariate, we tested several effects: the ego effect (individuals with high scores in a given attribute will groom more cows), the alter effect (individuals with high scores will receive grooming from more cows), and the homophily effect (individuals with the same or similar score will be more likely to groom one another). We added the effects one by one using a forward selection procedure, assessing for each effect the convergence of the model, the significance of the effect and the goodness of fit (GOF) statistic. We retained an effect if it was relevant for the study, did not affect convergence nor impaired GOF. We performed the estimation using the Method of Moments estimation procedure with 4 000 iterations. As required for an adequate convergence, T-ratios for deviations from targets need to be smaller than 0.1, and the overall maximum convergence ratio smaller than 0.25 (28). We assessed goodness of fit comparing the simulated networks to the observed network with regards to the following auxiliary network statistic: outdegree, indegree, triadic census, and geodesic distance distribution. The cumulative distribution of the simulated values is visualized using a violin plot and the observed values are represented by a superimposed solid red line (Supplementary Figure 1). Fit of auxiliary statistics is acceptable if the simulated values are similar to the observed values and fall within the 95% confidence interval defined in between two dotted gray lines. In addition, *p*-values > 0.05 indicate that the simulated values are close to the observed values.

**Table 2:** Description of the structural effects tested in the model.

**Structural effects**	**Description**	**Graphical representation**
Outdegree (density)	Tendency to groom other cows	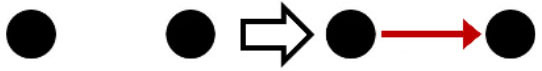
Reciprocity	Individuals tend to groom cows that previously groomed them	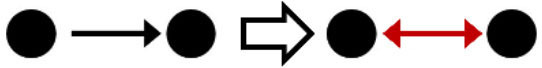
Three-cycle	Individuals tend to reciprocate grooming through triadic closure (generalized reciprocity)	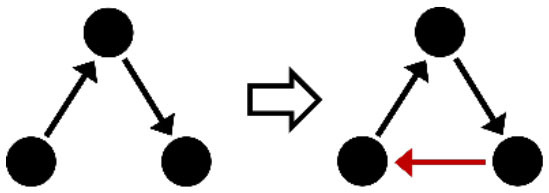
Transitive triplets	Individuals tend to groom cows with whom they share a common contact (triad closure)	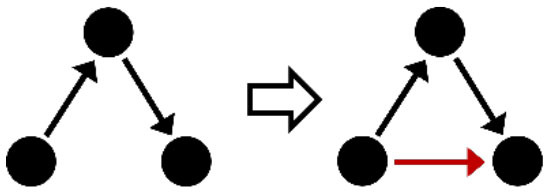
Transitive reciprocate triplets	Individuals tend to reciprocate grooming in triads	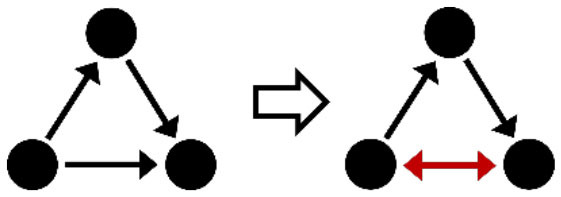
Indegree popularity	Individuals groomed by a larger number of cows—higher indegree scores—are more attractive to others and will to be groomed by even more cows on the next wave	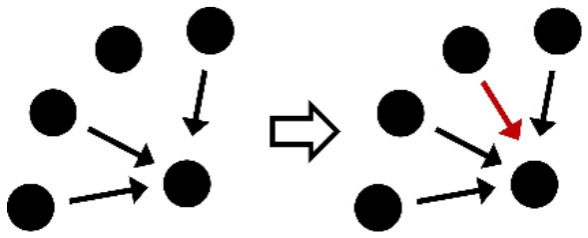
Outdegree popularity	Individuals that groom many cows—high outdegree scores—are more attractive to others and will be groomed by more cows on the next wave	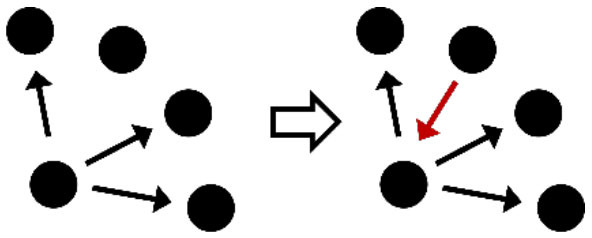
Outdegree activity	Individuals that groom many cows—high outdegree scores—will groom even more cows on the next wave	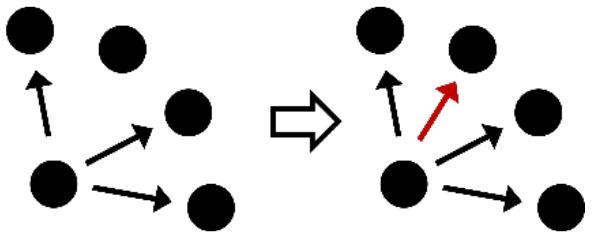
Out-outdegree assortativity	Individuals that groom many cows—high outdegree scores—will interact with cows with high outdegree scores	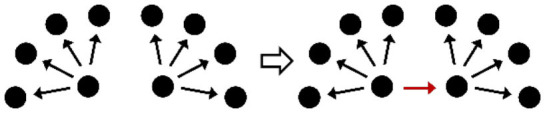

*Circles represent nodes, white arrows indicate the transition between two waves. Black arrows illustrate the interaction between nodes and red arrows correspond to new interactions*.

Rate parameters define the rate at which individuals that were not previously interacting form a new connection, or, on the contrary, individuals that were previously interacting stopped interacting. In that sense, we wanted to test if there were individual differences in how many changes they make in their outgoing ties, by evaluating the outdegree effect on rates change. We then tested the interaction between covariates and basic structural effects (i.e., reciprocity and transitive triplets). Also, to assess if reciprocity occurred mainly between individuals with similar attributes, we tested the interaction between reciprocity and homophily for different attributes. Before constructing the model, we examined the correlation matrix of the effects to detect high collinearity between parameters. Finally, we conducted time heterogeneity tests to inspect whether the parameters were stable across waves (28, 54).

## Results

We recorded a total of 1,329 allogrooming events from 38 cows during 30 experimental days. We excluded data from two individuals, one cow who died of hepatic failure in the third week of the study, and another cow whose calving was delayed and hence was observed for only 2 weeks.

**Figure 2:**
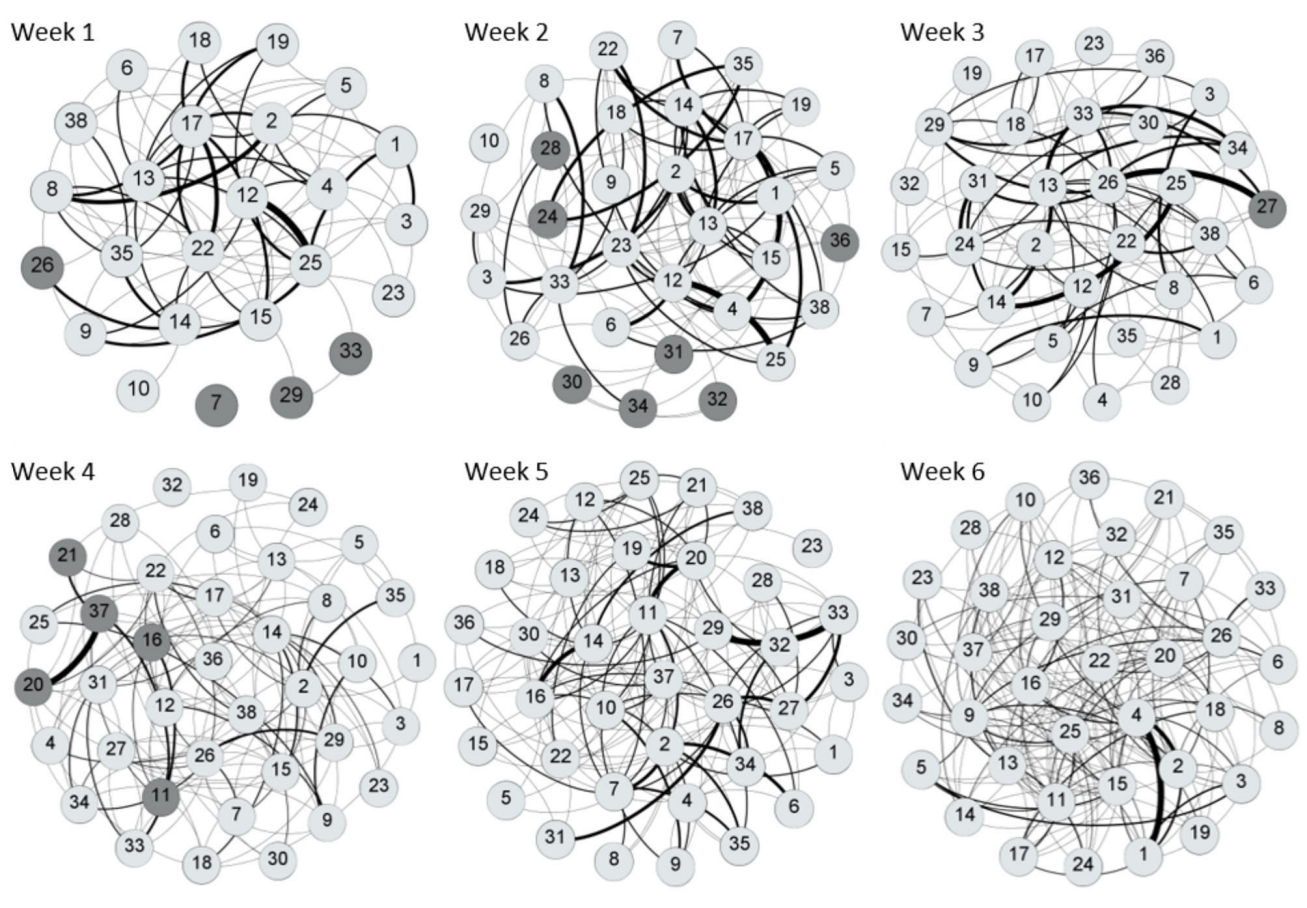
Visualization of the weekly social grooming networks using a Fruchterman Reingold layout. Nodes that are closer together exchanged more ties. Dark gray circles represent cows that were reincorporated to the group each week. Clear gray circles represent cows that were present in the group at least the week before. Tie width is related to the number of interactions, with wider ties indicating higher number of allogrooming bouts. The curve of the tie is turned clockwise, showing the direction of the interaction. Networks were obtained with Gephi 0.9.2 software (55).

**Figure 3:**
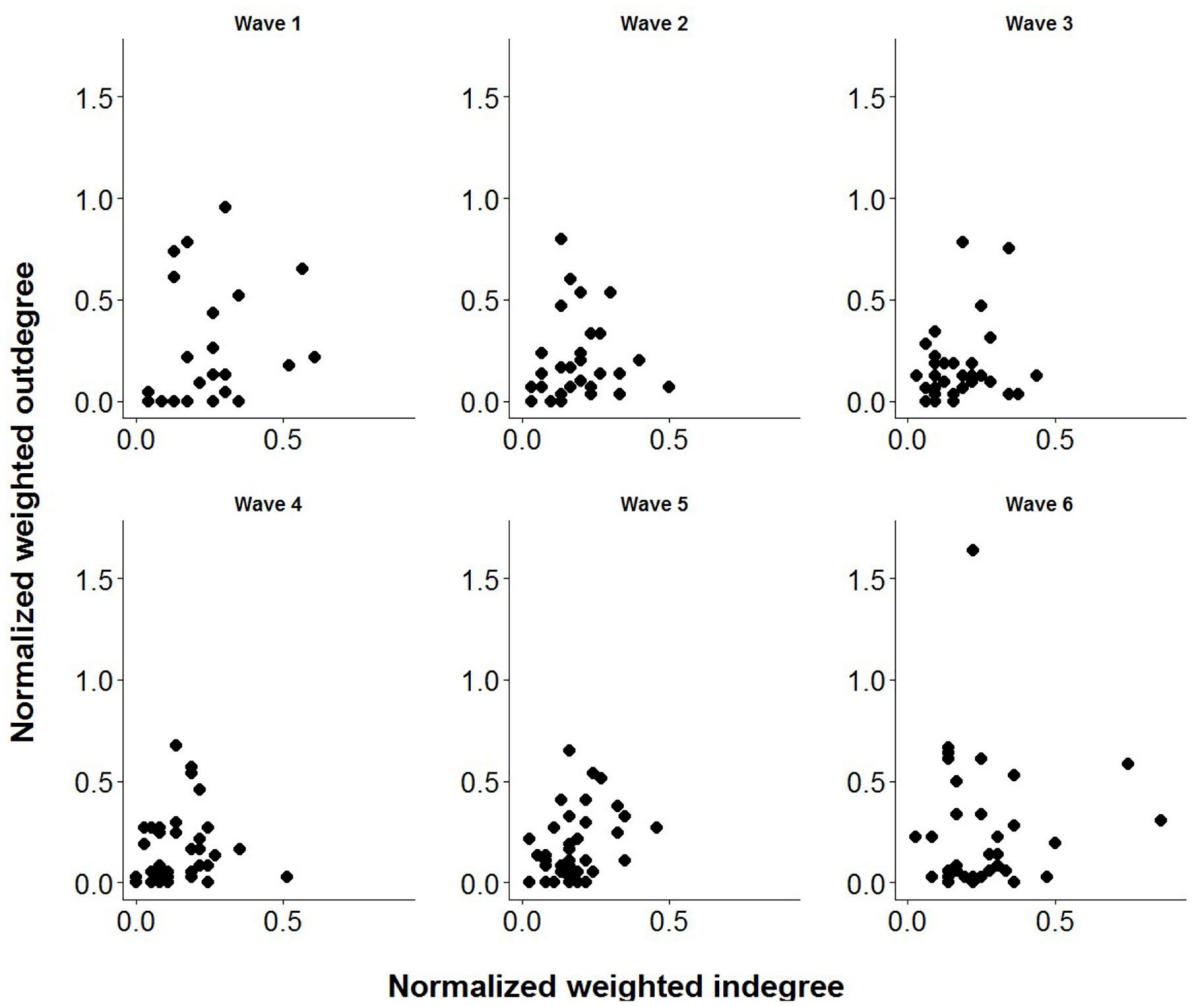
Association between normalized weighted outdegree and indegree values of the cows for each wave (week).

Tables 3, 4 provide information on descriptive statistics and dynamics of the network over the six waves, respectively. Density was medium to low, and network diameter and average path length were moderately large, evidencing a relatively sparse network. Figure 2 represents the allogrooming networks and Figure 3 displays the normalized weighted outdegree and indegree of each individual. Both figures indicate a considerable heterogeneity between cows in providing and receiving allogrooming.

**Table 3:** Descriptive statistics of network structure.

	**Measurement point**					
	**Wave**	**Wave**	**Wave**	**Wave**	**Wave**	**Wave**
	**1**	**2**	**3**	**4**	**5**	**6**
Number of nodes	25	32	33	38	38	38
Number of ties	148	187	180	218	252	344
Density	0.25	0.19	0.17	0.16	0.18	0.26
Network diameter (directed)	4	7	7	8	6	7
Average path length	2.10	2.87	2.85	2.92	2.50	2.54
Reciprocity	0.27	0.26	0.26	0.18	0.24	0.19

**Table 4:** Network dynamics.

	**Waves**				
	**1 → 2**	**2 → 3**	**3 → 4**	**4 → 5**	**5 → 6**
Cows entering the group	7	1	5	0	0
Cows leaving the group	0	0	0	0	1
Tie changes					
Creating tie (0 → 1)	81	74	99	120	142
Dissolving tie (1 → 0)	57	78	76	89	112
Stable tie (1 → 1)	36	39	37	47	55
Jaccard index	0.21	0.20	0.18	0.18	0.18

The final SAOM achieved convergence with an overall maximum convergence ratio of 0.19 and all t-ratios ≤ 0.10. Goodness of fit analyses indicated a reasonable fit of the model (Supplementary Figure 1). Time heterogeneity test was non-significant (χ^2^ = 2.44, d.f. = 4, *P* = 0.655), hence the parameter effects were stable across waves. We present the final model results in Table 5.

**Table 5:** Parameter estimates of the final model.

**Parameter**	**Estimate (standard error)**
Density	−1.965 (0.20)^***^
Outdegree effect on rate change	0.102 (0.04)^*^
Reciprocity	1.118 (0.14)^***^
Transitive triplets	0.133 (0.06)^*^
Transitive reciprocate triplets	−0.184 (0.11)
Outdegree popularity (sqrt)	−0.162 (0.05)^**^
Outdegree activity (sqrt)	0.233 (0.04)^***^
**Age**	
Alter	0.046 (0.03)
Ego	0.101 (0.02)^***^
Homophily	1.044 (0.18)^***^
**Social rank**	
Alter	−0.133 (0.05)^**^
Ego	−0.016 (0.05)
Homophily	0.259 (0.07)^***^
Same social rank x reciprocity	−0.319 (0.16)^*^
**Entry**	
Alter	0.119 (0.13)
Ego	0.328 (0.13)^*^
Homophily	0.096 (0.13)

* p < 0.05;

** p < 0.01;

*** *p < 0.001*.

We found a negative density parameter, indicating that cows did not groom every other cow. Moreover, the positive reciprocity effect indicate that cows were more likely to groom cows that had previously groomed them. The model also indicated a significant transitive triplet effect, indicating triadic closure. Therefore, having a common contact increased the probability of contact. The absence of transitive reciprocate triplets implies that even though cows do form transitive triplets, they do not reciprocate grooming in these closed triads. The negative outdegree popularity effect revealed that cows with high outdegree, meaning those that were more active groomers, tended to be groomed by fewer cows over time. Additionally, we found a positive outdegree activity, suggesting that cows that groomed a high number of cows, continued doing so or groomed even more cows over time. The model indicated a positive interaction between rate parameters and outdegree effect, reflecting that highly active social groomers contributed more to changes in tie patterns than the remaining cows. A positive age ego indicated that older cows groomed more individuals than younger cows. We also found a negative SR alter, suggesting that individuals with higher social rank were groomed by fewer cows over time. The model displayed a positive age and SR homophily, identifying a strong preference for grooming similar-age and ranked cows. However, the negative interaction between SR and reciprocity indicated that the more similar the social rank was between two cows, the less probable they were to reciprocate grooming. In addition, the positive Entry ego suggested that individuals introduced to the group after the beginning of the observation period groomed more cows through time than the remaining cows. Reproductive state effects were not significant and did not improve the model, hence we excluded this parameter from the analysis.

## Discussion

This study explored the dynamics of the social grooming network in a group of dairy cows in early postpartum using stochastic actor-oriented models. Our findings show that structural processes and individual attributes determine social grooming activity in the group. There was a substantial amount of variability in the intensity of performing but also receiving allogrooming, indicating individual differences among cows. Some cows were very active and central in the network, while others almost did not interact. Previous studies on social grooming in cattle found similar results (17, 47, 48, 56), suggesting that specific individuals might be key players in the network structure.

Overall, the network was sparse, holding only 16 to 26% of all the possible dyadic interactions that could have existed. This is supported by the negative density effect in the final model, reaffirming that cows groomed a limited number of individuals. Sparseness is common in animal contact networks (19), as individuals normally interact with only a fraction of all the members of their community, especially in species that have some level of social structure (57). The probability of an interaction will depend on factors such as attributes of each individual, past experience and group and environmental factors (58). Sparseness tended to increase—indicated by a drop in density and an increase in diameter—in week 2 when several cows entered the group. As cows continued to enter the group, sparseness continued to increase until week 5 when no new cows were added. At that moment, network density started to rise, and returned to week 1 value at week 6, when the group was stable. Additionally, although stable ties tended to increase in the last 2 weeks when no new cows were added to the group, the low Jaccard index suggests that there was a considerable reorganization of ties from 1 week to the next, with only 18 to 21% of the ties persisting each time. However, these changes were probably minor since the time heterogeneity test of the model was non-significant. Therefore, even though there were some changes in network parameters over the weeks, the network was relatively stable. Some instability could be expected, considering that those cows had just been regrouped. Even though cows' relationships and hierarchy are stable and can persist for years without being affected by regroupings (59, 60), it is important to note that those cows were recently separated from their former group and from their calf, were just recovering from calving and were under important metabolic and hormonal changes. It is therefore understandable that they needed some time to readjust to this new situation, especially for primiparous cows.

In different species, there is evidence that allogrooming could serve different social functions, which might also be true for cattle. One possible function is *cooperation by reciprocity of allogrooming*. In this form of cooperation, individuals alternate their roles in such a way that both benefit over time (61). Social grooming is mainly directed toward the head and neck of other cows (17, 62), body parts that are difficult to reach by the animals themselves. Therefore, cows might groom each other as a form of cooperation, by reciprocating grooming (exchange grooming received for grooming given). Our model results showed a significant reciprocity effect (at dyadic level), reflecting an increasing tendency for cows to groom other cows that had previously groomed them. However, at the group level, only 18 to 26% of the grooming events were reciprocated, which is in line with results reported by Val-Laillet et al. (17). We also found that reciprocity was mainly performed between individuals of different social ranks. It seems that reciprocity, although important, is not a predominant aspect of social grooming patterns and might only be performed by specific types of individuals.

Allogrooming could also be used as a *rank-related currency*. It has been proposed that lower rank individuals exchange grooming for other benefits such as access to food or protection (58, 63, 64). We would therefore expect this behavior to be mainly performed by lower-ranked individuals toward higher-ranked individuals, trading it with social benefits such as tolerance around food or lower aggression. We found that while the social rank of a cow did not influence how many cows she groomed, it did influence how many individuals she received grooming from. Specifically, high-ranking cows were licked by significantly less individuals over time than the rest of the cows. The literature shows controversy over the relationship between social rank and allogrooming in cattle. While some of our results are in line with previous studies in that the number of cows groomed by an individual was independent of its social rank (16, 17, 62), other studies found different results. Reinhardt et al. (15) observed low-ranking adult individuals performing social licking more often than high-ranking individuals. In contrast, Šárová et al. (48) found that higher ranking cows performed more allogrooming than subordinate cows, and that this behavior was mostly oriented down the hierarchy. It is important to bear in mind that those discrepancies may be due to differences in study design, cattle type, and rearing system. Moreover, there is still no consensus on the most appropriate method to determine social rank in dairy cows, and the complexity of agonistic interactions in this species has been stressed earlier (5, 65). This would explain the considerable inconsistencies in the methods used to determine social rank in previous studies and could be the source of variation contrasting the results.

Finally, allogrooming could also serve to maintain g*roup stability and cohesiveness*. Šárová et al. (48) proposed that dominant cows maintain herd cohesiveness by being more active allogroomers. While we did not find evidence that higher-ranked cows groomed more individuals, we did find that older cows were more active groomers. In cattle, hierarchy is strongly related to the age since females born in a farm usually stay in that farm all their life (66–68). We therefore speculate that older cows performed more allogrooming to maintain herd stability and cohesiveness, aspects that are important for gregarious and social species like cattle.

We found a strong age homophily effect, indicating an increased preference over time for grooming cows of similar age. There is evidence that allogrooming is strongly related to growing up together, which denotes familiarity between individuals (5, 16). Calves that were raised together for at least the first 6 months of their lives formed preferential relationships that lasted for several years (4). Those bonds are illustrated by increased spatial proximity, synchronization of activities, and affiliative behaviors (12, 17, 41). This age-assortativity effect suggests that, despite being separated numerous times during their productive life, cows maintain these preferential relationships. A period of readjustment may occur when they meet again after separation, as it happened during the early postpartum period, but after they became reacquainted there was an increased preference for grooming cows of similar age. This preference might also explain the significant social rank homophily present in the final model. As we mentioned previously, social rank is strongly related to age, thus the increased preference for grooming cows of similar social rank might in fact be explained by age homophily (13).

Cows that had been recently reintroduced to the group tended to groom more cows than the rest of the herd. As these behaviors reinforce bonds and reduce tension, new cows might have engaged in more allogrooming to increase tolerance and avoid aggression, facilitating their acceptance into the herd. On the other hand, cow-calf separation may have also affected the expression of the licking behavior. These cows had calved less than 24 h prior to being brought into the study area and had just been separated from their offspring. Calf licking is a primary maternal behavior that facilitates the cow-calf bond (69), and in absence of the calf, cows might have redirected the expression of this behavior toward other members of the herd. As the underlying motivation of those cows to groom more individuals is not clear, it would be beneficial to remove the effect of the separation from the calf; for example, evaluating allogrooming patterns after regrouping at another stage of the productive cycle.

Oestrus had no impact on the dynamics of allogrooming. This supports previous findings that reproductive status does not affect stable relationships in cows (59). Oestrus is a brief event, lasting about 14 h (70) and occurring approximately every 21 days between one pregnancy and another (71). Therefore, it probably does not have a long-term impact on cow relationships or interactions between cows.

Results indicated a significant transitive triplets effect, representing triad closure. In other terms, if a cow A groomed a cow B, and B groomed a cow C, then A was more likely to groom C subsequently (“the friend of my friend is also my friend” effect). This effect might be related to proximity, as cows frequently interacting with each other also spend more time in near one another (5). There is therefore an increased chance that if both cows A and C spend more time in proximity of B and interacting with her, they will end up interacting themselves, explaining the closure of the triad.

Very active groomers changed their relations faster over time, meaning that they were less consistent in their choice of recipient. They also tended to groom more cows over time, even though their popularity was declining (i.e., they were groomed by fewer cows). This effect could not be explained simply by social rank, age or other individual characteristics, as there was an absence of correlation between those effects and outdegree effect on rate (Supplementary Figure 2). We speculate that those patterns could represent roles that individual cows undertook (3). As allogrooming enhances bonding and group cohesion, some cows might have assumed the role of maintaining group stability. It could also be related to different personality traits, however, long term studies are needed to explore this possibility.

Our study has several limitations which should be considered when interpreting our results. First, this study was performed with only one group of dairy cows and therefore need to be replicated. Furthermore, allogrooming patterns are probably influenced by factors such as group composition, in relation to social rank and age distribution, and the resources available. Thereby, the results need to be supported with further research on different types of herds, to extend results to a broader population with more conclusive findings. Second, another aspect that would have strengthened the findings would have been to use a stable group to compare network patterns. However, this study was performed under commercial conditions, and herds in those systems are not stable, given that grouping and regrouping is constant year round. Furthermore, performing social network studies is logistic and resource-costly. With new technological advances, such as spatial proximity loggers, far more information can be collected, although some doubts still persist over the reliability of the data (72). Still, direct observation has the advantage of providing very precise information about the type and direction of the contact, something telemetry cannot provide at present time. Finally, it would have been interesting to include in the model how many periods of their productive life did the cows shared, to see how much that factor influenced grooming patterns.

## Conclusions

This study provides further insights on the dynamics of allogrooming in dairy cows. Overall, the network was stable, suggesting that the cows' grooming patterns were consistent despite the weekly incorporation of individuals. We found considerable individual differences, with some cows more active and central in the network than others. This variability in grooming patterns was influenced by age, familiarity, social rank, and moment of reintroduction to the group. Those patterns might reflect complex underlying processes such as cooperation by reciprocity of allogrooming, and also the presence of roles some cows might undertake to maintain group stability and cohesiveness. Future studies could continue to enhance our understanding of these processes and the dynamics of cattle interactions during regrouping, for example by comparing prepartum and postpartum networks. It would be also very interesting to compare the effects of different modifications in group composition, for example how many cows are incorporated each time and the characteristics of those cows.

This study highlights the usefulness of considering social network dynamics to understand the complexity of cows' relationships. Improving our knowledge on social grooming patterns, together with other social behaviors, will allow the design of management practices that are more integrated with cows' social behavioral needs.

## Data Availability Statement

The datasets generated for this study are available on request to the corresponding author.

## Ethics Statement

The animal study was reviewed and approved by University Austral of Chile's Animal Care Committee (protocol number 252/2016). Written informed consent for participation was not obtained from the owners because it is not needed a written informed consent in Chile.

## Author Contributions

IF, GM, and AS contributed to the conception and design of the study, the collection of the data, and the statistical analysis. IF, GM, AS, and JP substantially contributed to the discussion of the results, provided critical feedback, and contributed to the writing of the manuscript. All authors contributed to the article and approved the submitted version.

## Conflict of Interest

The authors declare that the research was conducted in the absence of any commercial or financial relationships that could be construed as a potential conflict of interest.
