# Supplementary material for: Understanding Allogrooming Through a Dynamic Social Network Approach: An Example in a Group of Dairy Cows
Source: Front Vet Sci. 2020 Aug 4;7:535. doi: 10.3389/fvets.2020.00535 (PMC7417353; doi:10.3389/fvets.2020.00535)
Supplement: Supplementary file 1 [file Data_Sheet_1.docx]

***Supplementary Material***


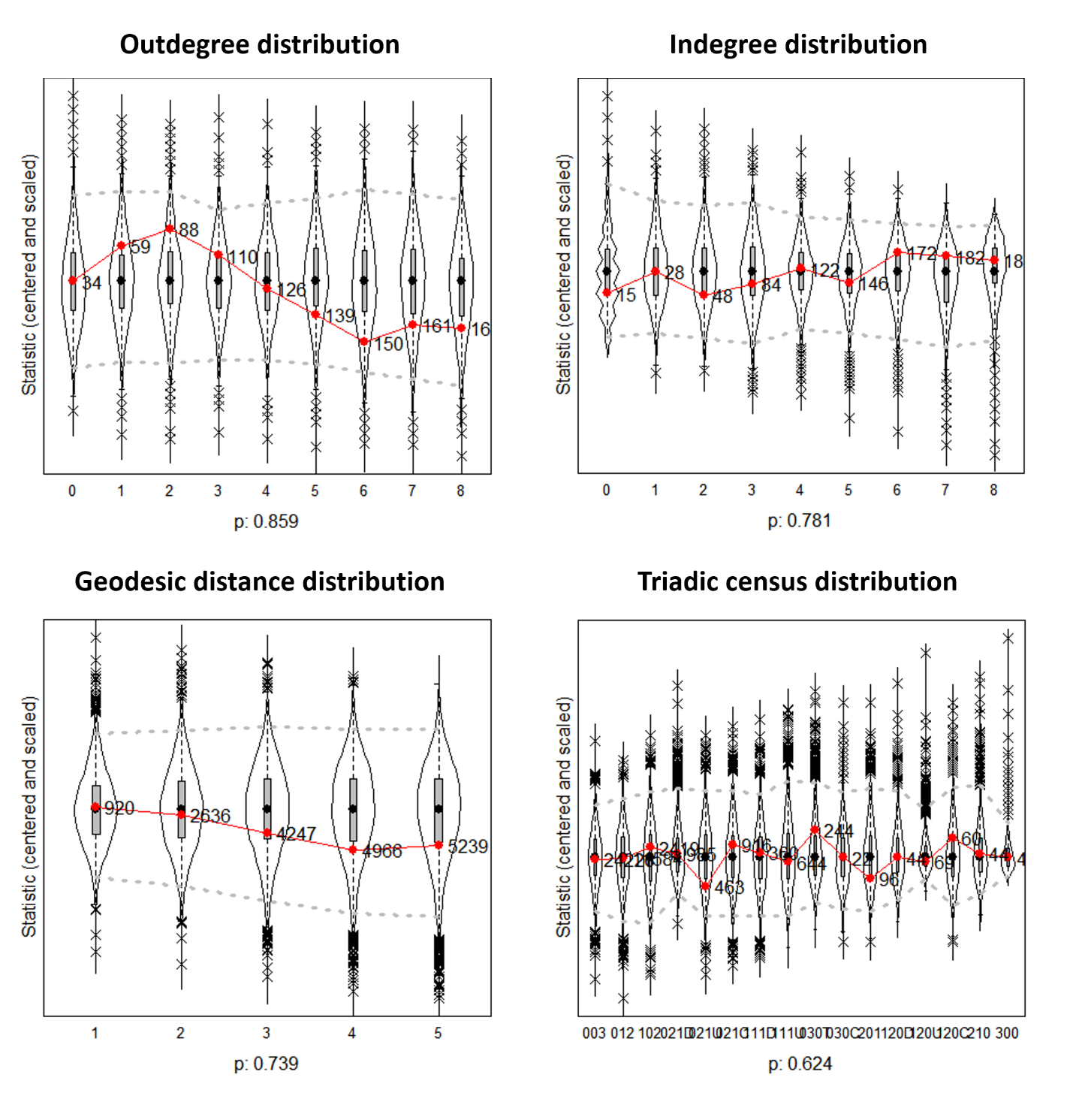


**Supplementary Figure 1.** Goodness of fit results of auxiliary network statistics (outdegree, indegree, geodesic distance, triadic census). Violin and boxplots represent the cumulative distribution of the simulated values, and the solid red lines indicate the observed values. The dotted gray lines show the 95% confidence intervals. Fit of auxiliary statistics is acceptable if the simulated values fall within this interval, and the p-values are > 0.05, indicating that the simulated values are close to the observed values.

**Supplementary Figure 2.** Effects correlations
